# Sphingosine‐1‐Phosphate Lyase Inhibition Increases Glycolysis in Adult Cardiomyocytes and Restores Glycolytic Flux in Diabetic Cardiomyopathy

**DOI:** 10.1111/jcmm.70924

**Published:** 2025-11-03

**Authors:** Jens Vogt, Melissa Kim Nowak, Marcel Benkhoff, Hao Hu, Philipp Wollnitzke, Lisa Dannenberg, Amin Polzin, Bodo Levkau

**Affiliations:** ^1^ Institute of Molecular Medicine III University Hospital Düsseldorf, Heinrich Heine University Düsseldorf Düsseldorf Germany; ^2^ Department of Cardiology, Pulmonology, and Vascular Medicine, Medical Faculty Heinrich Heine University Düsseldorf Düsseldorf Germany; ^3^ Cardiovascular Research Institute Düsseldorf (CARID), Medical Faculty Heinrich Heine University Düsseldorf Düsseldorf Germany

**Keywords:** cardiomyocyte, diabetic cardiomyopathy, glycolysis, hypertrophy, metabolism, sphingosine‐1‐phosphate

## Abstract

Sphingosine‐1‐phosphate (S1P) is a bioactive lipid that affects cardiac contractility and calcium homeostasis and exerts potent cardioprotective properties in myocardial infarction, heart failure, preconditioning. Whether and how it may affect energy metabolism in the heart is still unknown. Here, we examined S1P effects on glycolysis of adult cardiomyocytes (ACM) using Seahorse technology and observed that intracellular S1P rather than extracellular S1P potently potentiates basal glycolysis and increases glycolytic capacity. Accordingly, ACM from mice administered a S1P lyase inhibitor to prevent S1P degradation featured 3‐fold higher S1P levels and a 30%–40% increase in basal glycolysis and glycolytic capacity, whereas acute S1P stimulation had no effect. Cardiomyocyte‐specific GLUT4‐deficient ACM were resistant to this increase, whereas ACM from S1P lyase‐inhibited mice featured a 3‐fold higher glucose uptake, suggesting that higher glycolysis may be a function of increased glucose influx through GLUT4. Comparing glycolysis in ACM from normal chow‐fed mice with ACM from pre‐diabetic mice following long‐term feeding of a high caloric diet revealed a rapid and progressive loss of glycolytic potential without yet affecting cardiac function despite a beginning hypertrophy on echocardiography. Most importantly, both could be reconstituted to normal by S1P lyase inhibition. As the levels of bioactive lipids such as S1P are altered in obesity and diabetes, understanding their effects on metabolism may help reveal novel aspects of lipid biology in metabolic diseases of the heart.

**Abbreviations**
2‐DG: 2‐deoxy‐d‐glucose
2DG6P: 2‐DG 6‐phosphate
AA: antimycin A
ACM: adult cardiomyocyte
DIO: diet‐induced obesity
DOP: 4‐deoxypyridxone
ECAR: extracellular acidification rate
EDV: end‐diastolic volume
EF: ejection fraction
ESV: end‐systolic volume
FA: fatty acid
Glc: glucose
GLUT: glucose transporter
GTT: glucose tolerance test
HFD: high‐fat diet
IVS: intraventricular septum
MRM: multiple reaction monitoring
Rot: rotenone
S1P: sphingosine‐1‐phosphate
S1PR: S1P receptor

## 1 Introduction

The heart has an enormous demand for energy and belongs to the most metabolically active organs [1, 2, 3]. The vital mechanical work is performed through the constriction and relaxation of cardiomyocytes that make up the largest volume of the ventricular wall [4, 5, 6, 7]. To satisfy the constant energy demand, adult cardiomyocytes generate ATP mostly from mitochondrial oxidative phosphorylation using fatty acids (FA) as the main and most potent energy source. However, cardiomyocytes also utilise other substrates such as carbohydrates, lactate, amino acids, ketone bodies for energy production. This ability to metabolise diverse classes of substrates enables the heart to be metabolically flexible and ensures sufficient ATP production despite fluctuating availability of substrates and/or sudden changes in energy demand. This is essential for the heart, as the high turnover but low ATP content of 8–10 mM requires its constant ATP production to maintain cardiac function [8, 9].

The importance of this metabolic flexibility becomes apparent during pathological conditions such as myocardial infarction, cardiac hypertrophy, and diabetes, when cardio‐metabolic balance is disrupted and prolonged shifts of substrate preference occur. The two main shifts during pathophysiological conditions are either an overreliance on beta‐oxidation with reduced glucose utilisation, as in the diabetic heart, or persistently increased glycolysis, as commonly noted in cardiac hypertrophy and heart failure [10, 11, 12, 13, 14, 15, 16, 17, 18, 19, 20]. These chronic shifts in cardiac metabolism are the result of pathophysiological changes in the regulation of transporters and enzymatic shifts used to fine‐tune the substrate utilisation—the so‐called Randle cycle. The Randle cycle, also termed ‘glucose‐fatty acid cycle’, is used to regulate the metabolic competition between glucose and FAs as substrates for energy generation [21]. In ACMs, FAs oxidation causes a decrease of glucose utilisation by inhibiting glucose uptake and catabolism. Several FA oxidation intermediates such as citrate and FA‐acyl CoA suppress glucose utilisation by inhibiting glucose transporters (GLUT) and key glycolytic enzymes such as hexokinase, phosphofructokinase, pyruvate dehydrogenase [22, 23, 24, 25, 26, 27, 28]. Even a short‐term feeding of high‐fat diet (HFD) or direct exposure to lipids is capable of repressing glucose uptake [24, 29, 30] by inhibition of the insulin‐stimulated AKT activation that promotes translocation of GLUT4 from the intracellular storage to the cell membrane to facilitate glucose uptake [31]. Chronic overexposure to lipids as in obesity or diabetes and the concomitant increase of intracellular intermediates result in a dysregulation of these molecular mechanisms through negative feedback and adaptation. One such maladaptation is the heart's desensitisation to insulin through dampening of AKT signalling [32, 33] and decreased expression of GLUT4 [22, 30].

However, many of these studies have been performed in isolated perfused hearts or in vitro in cell lines, and only a few have addressed energy metabolism at the single adult cardiomyocyte level using extracellular flux technology. Instead, mostly neonatal cardiomyocytes or myocytic cell lines have been employed, which differ considerably in transcriptomics, proteomics, lipidomics, and most certainly, metabolomics from ACM [34, 35, 36, 37, 38]. Also, no study so far has compared glucose metabolism in adult cardiomyocytes from normal and insulin‐resistant diabetic or pre‐diabetic hearts. Here, we have examined how overall metabolic changes induced by HFD in vivo, such as hyperglycaemia and insulin resistance, and in vitro models recapitulating these changes correlate with and contribute to energetic disturbances in adult mouse cardiomyocytes.

Moreover, we have examined how altering the metabolism of sphingosine‐1‐phosphate (S1P), a bioactive lipid with potent cardioprotective properties in myocardial infarction, heart failure and preconditioning [39, 40, 41, 42] may affect energy metabolism in healthy and diabetic cardiomyocytes. The rationale for looking into this was our recent discovery that non‐S1P receptor‐mediated intracellular S1P signalling dynamically regulated GLUT1‐ and GLUT4‐mediated glucose uptake in red blood cells under normal and diabetic conditions [43].

## 2 Material and Methods

### 2.1 Ethics Statement

All mouse experiments were performed according to ARRIVE (Animal Research: Reporting of In Vivo Experiments) 2.0 guidelines and were approved by the State Office for Nature, Environment and Consumer Protection (LANUV) of North Rhine‐Westphalia (Germany), in accordance with the European Convention for the Protection of Vertebrate Animals used for Experimental and other Scientific Purposes.

### 2.2 Mouse Model

Male mice were used. C57BL/6J mice were obtained from Charles River Laboratories or purchased from Janvier Labs (Saint‐Berthevin, France). α‐*MHC*‐*Cre GLUT4 loxP* mice were originally a kind gift from Abel, Department of Medicine, David Geffen School of Medicine, University of California, Los Angeles, CA, USA, as published previously [44]. All mice were housed and cared for within the central animal research facility of the Heinrich Heine University Düsseldorf at an ambient temperature (22°C) on a 12/12 h light/dark cycle with ad libitum drinking water. Normal chow animals were fed a standard rodent diet. DIO mice were fed a high‐fat diet (60% kcals from fat) for 12 or 16 weeks. To inhibit the sphingosine‐1‐phosphate lyase, 4‐deoxypyridoxine (DOP, Sigma‐Aldrich: D0501) was administered via drinking water for either 2 weeks in normal chow animals or for 6 weeks in DIO mice before being sacrificed.

### 2.3 Glucose Tolerance Test

Glucose tolerance tests (GTT) were performed on overnight fasted mice. Blood was drawn from the tail vein and blood glucose concentration was determined (0 min) using the StatStrip Xpress2 glucometer (Nova Biomedical, USA). Blood glucose levels were subsequently measured at 15, 30, 60, 90, 120, 240 min after intraperitoneal glucose injection (2 g/kg body weight).

### 2.4 Isolation of Primary Adult Cardiomyocyte

Adult cardiomyocytes (ACM) were isolated from mice by the Langendorff perfusion method. Briefly, the mice were sacrificed by cervical dislocation, and the heart was rapidly excised and placed in ice‐cold Tyrode buffer (11.3 mmol/L sodium chloride, 0.47 mmol/L potassium chloride, 0.06 mmol/L potassium dihydrogen phosphate, 0.06 mmol/L disodium hydrogen phosphate, 0.12 mmol/L magnesium sulphate, 1.2 mmol/L sodium hydrogen carbonate, 1.0 mmol/L potassium hydrogen carbonate, 1.0 mmol/L HEPES, 3 mmol/L taurine, 10 μmol/L blebbistatin Biozol: APE‐B1387 and 5.5 mmol/L glucose). To facilitate retrograde perfusion, the aorta was cannulated with a blunted 18‐gauge needle under a stereo zoom microscope and secured in place with silk suture. The cannulated heart was then attached to a simplified Langendorff apparatus and perfused with 37°C‐warm Tyrode buffer (with 0.01 mmol/L calcium chloride, pH 7.42) for 3 min. Afterwards, the system was switched to an enzyme solution (37°C Tyrode with 0.01 mmol/L calcium chloride, pH 7.42+ 0.014% Trypsin EDTA (Gibco: 15090046) and 0.075 mg/mL Liberase TM (Merck: 5401127001)) for 5 min. After digestion, all other tissue (e.g., aorta, atria, fatty tissue) was carefully removed from the ventricles and discarded. The ventricles were transferred to a dish with 3 mL enzyme solution and pulled apart with fine forceps. The remaining tissue was gently triturated using a 1 mL pipette with cut‐off tip end and filtered through a 200 μm polyamid sieve cloth (neoLab: 4‐1413). The enzymatic digestion was quenched through the addition of 3 mL warm Tyrode buffer containing 20% FCS. The ACM cells were allowed to sediment by gravity in a round‐bottom 14 mL tube for 10 min at room temperature. After sedimentation, the solution was aspirated, and the pelleted cells were repeatedly gently re‐suspended and centrifuged in solutions with increasing calcium concentration (0.1 mmol/L, 0.2 mmol/L, 0.4 mmol/L, and 0.8 mmol/L calcium chloride). Lastly, after the final of the four calcium gradients was complete, the solution was carefully aspirated, and the ACM were re‐suspended in plating medium (M199 medium (Hanks' Balanced Salts, Gibco: 12350039) containing 10% FCS, 1×Antibiotika‐Antimykotikum (Gibco: 15963194), 1×Insulin‐Transferrin‐Selen (Gibco: 12097549), 10μmol/L blebbistatin; 5 mmol/L creatine, 2 mmol/L l‐carnitine, 5 mmol/L taurine). This method produced ≈5 × 10^5^ viable rod‐shaped cells per heart.

### 2.5 Seahorse Analysis

Determination of real‐time changes in cellular bioenergetics measurements of extracellular flux rates using a Seahorse XF Pro Analyser (Agilent Technologies, Santa Clara, USA) was used according to manufacturer's instructions, with minor optimisations.

Prior to the isolation, each well of the Seahorse XFe96 tissue culture plate was coated with 50 μL of a 10 μg/mL mouse laminin (Merck: CC095‐5MG‐M) solution overnight at 4°C, followed by incubation at 37°C for 2 h. Immediately before seeding the cells in plating medium, the laminin solution was removed from the plate. Cells were seeded at a density of 175 cells/mm^2^. The ACM were cultivated at 37°C in the presence of 5% CO_2_ in a humidified incubator for 1 h after isolation. Two hours before the assay, the plating medium was replaced with XF DMEM (Agilent: 103575‐100) supplemented with 1× Antibiotic‐Antimycotic, 1× Insulin‐Transferrin‐Selenium, 10 μmol/L blebbistatin, 0.5 mmol/L l‐carnitine, 2 mmol/L l‐glutamine. For wells designated for the Glycolytic Rate Assay, an additional 10 mmol/L glucose was added. The same medium was maintained throughout the measurement.

The measurements were controlled by an automated programmable protocol which consists of microplate insertion, measurement of baseline extracellular acidification rate (ECAR) in 180 μL XF DMEM followed by injections of substrates or inhibitors and repeated measurements of ECAR. The injections for the Seahorse Glycolytic Rate assay consist of a mixture of rotenone (Rot, Adipogen: AG‐CN2‐0516‐G005) and antimycin A (AA, LKT Laboratories: A5378) to an end concentration of 1 mmol/L each and 50 mmol/L 2‐deoxy‐d‐glucose (2‐DG, Sigma‐Aldrich: D6134). The Seahorse Glycolysis Stress assay is performed in glucose‐free media followed by sequential addition of 10 mmol/L glucose (Glc, Agilent: 103577‐100), Rot/AA and 2‐DG.

### 2.6 Glucose Uptake‐Glo Assay Uptake

Cellular glucose uptake was measured indirectly using the Glucose Uptake‐Glo assay (J1341, Promega). Briefly, the kit uses 2‐DG as a glucose analog, which is transported into cells and converted to 2‐DG 6‐phosphate (2DG6P) by hexokinase. 2DG6P cannot be further metabolised and is quantified by an enzymatic luminescence reaction.

The assay was performed as per the manufacturer's instructions with slight modifications to account for the specificity of ACMs. Briefly, freshly isolated ACMs were seeded in 96‐well plates (5 × 10^3^ cells/well) and cultivated for 1 h. Medium was aspirated and exchanged for glucose‐ and phenol red‐free medium. The ACMs were then incubated for 2 h at 37°C with 5% CO_2_. Afterwards, 2‐DG was administered to a final concentration of 1 mM for 20 min. Stop buffer was added, and the plate was shaken vigorously at room temperature for 30 min. Then, neutralisation buffer was added and the plate was shaken vigorously before detection reagent was added to each well and the plate was incubated at room temperature for 90 min. Afterwards, the solution was transferred into a white 96‐well plate, and luminescence was recorded using a CLARIOstar Plus microplate reader (BMG LABTECH GmbH, Offenburg, Germany).

### 2.7 LCMS Measurements

S1P measurements were performed as described [43]. In brief, chromatographic separation for S1P was performed with a 2 × 60 mm MultoHigh‐C18 RP column with 3 μm particle size at 40°C on a LCMS‐8050 triple quadrupole mass spectrometer (Shimadzu Duisburg, Germany) interfaced with a Dual Ion Source and a Nexera X3 Front‐End‐System (Shimadzu Duisburg, Germany). MS settings were the following: Interface: electrospray ionisation, nebulising gas flow: 3 L/min, heating gas flow: 10 L/min, interface temperature: 300°C, desolvation temperature: 526°C, DL temperature: 250°C, heat block temperature: 400°C, drying gas flow: 10 L/min. Flow rate was 0.4 mL/min. Mobile phases for S1P measurement consisted of [A] = methanol and [B] = aqueous HCO_2_H (1% vol/vol) and the following gradient settings were used: [A] increased from 10% to 100% over 3 min (B.curve = −2) and returned to 10% from 8.01 min to 10 min prior to the next injection. Data were collected using multiple reaction monitoring (MRM) and positive ionisation was used for qualitative analysis and quantification. Standard curves were generated by measuring increased amounts of analytes (100 fmol to 50 pmol S1P) with internal standard (C_17_‐S1P = 1 pmol or S1P d:18:1‐d_7_ = 1 pmol). Injection volume of all samples was 10 μL and the following MRM transitions (positive mode) were used for quantification: *m*/*z* = 380.3 → 82.0, 264.1, 247.2, 362.2, 282.3 for S1P, *m*/*z* = 366.2 → 250.1, 348.2, 268.3, 233.3 for C_17_‐S1P and *m*/*z* = 387.2 → 271.25, 254.3, 369.45, 81.9 for S1P‐d_7_. Linearity of standard curves and correlation coefficients were obtained by linear regression analysis. All MS analyses were performed with LabSolutions 5.114, analysed with LabSolutions Insight (Shimadzu, Kyoto, Japan) and further processed in Microsoft Excel.

### 2.8 Echocardiography

Echocardiography was performed as established [40]. In brief, mice were anaesthetised with isoflurane (1.5 vol%) and laid on a warming pad to keep body temperature stable at 37°C–38°C. Ultrahigh frequency Visualsonics Vevo 3100 (Fujifilm) with a high‐resolution ultrasound transducer (18–38 MHz) was used to gain images and videos in the parasternal long‐axis view. Measurements and post‐processing analyses were performed with commercial software (VevoLab 3.2.6., Visualsonics).

### 2.9 Statistics

All data are expressed as the mean ± SD. Statistical analyses for two groups were carried out using a Mann–Whitney *U* test for multiple comparisons or continued measurements 2‐way ANOVA with Šídák correction. *p*‐value < 0.05 was considered statistically significant. All statistical analyses were performed using GraphPad Prism 9 (GraphPad Software).

## 3 Results

### 3.1 Short‐Term Sphingosine‐1‐Phosphate Lyase Inhibition In Vivo Stimulates Glycolysis and Glucose Uptake in ACM

**FIGURE 1:**
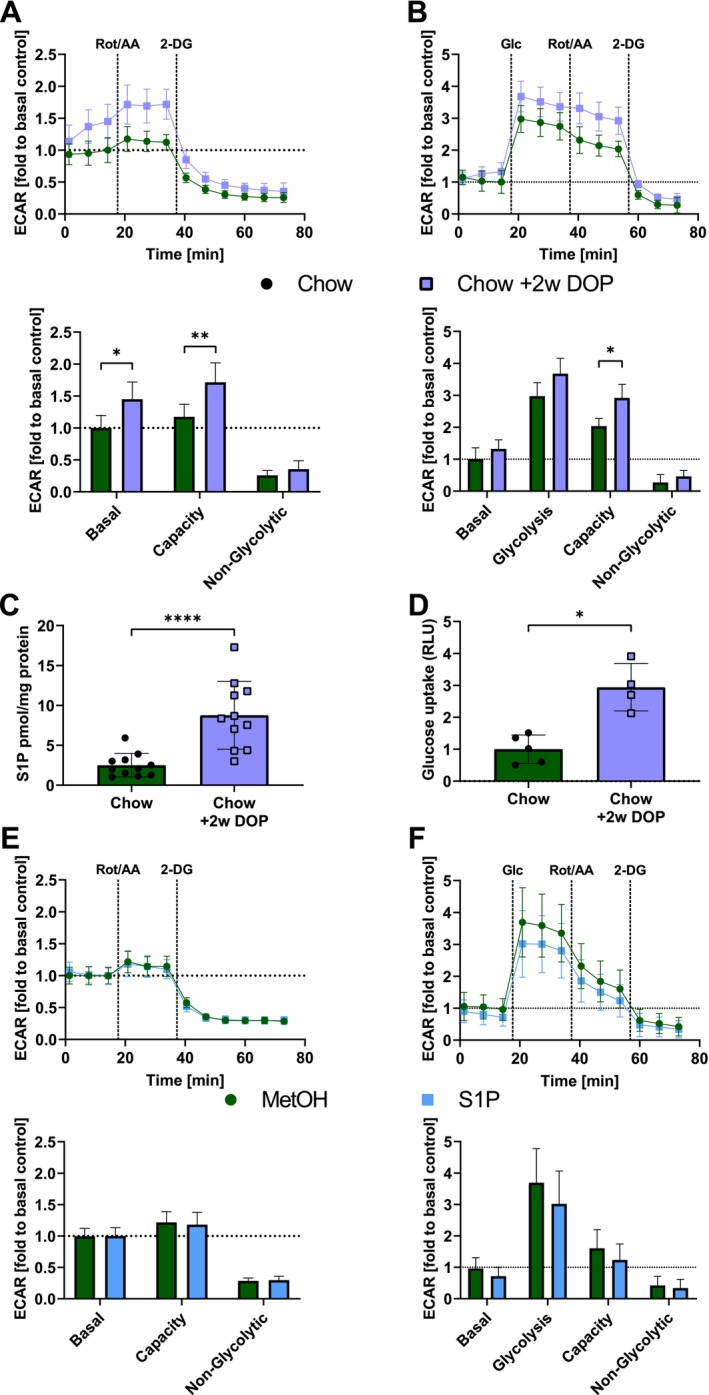
Glycolysis is improved in ACMs with in vivo inhibition of sphingosine‐1‐phosphate lyase but not after acute in vitro S1P stimulation. Representation of relative extracellular acidification rates (ECAR, 1 = control basal) of cardiomyocytes from controls and DOP treated mice during (A) XF Seahorse Glycolytic Rate and (B) Glycolysis Stress assays. At the indicated times glucose (Glc, 10 mM), a rotenone and antimycin A mix (Rot/AA, 1 μM each) or 2‐deoxy‐d‐glucose (2‐DG, 50 μM) were injected. Each tracing shows the calculated weighted mean and standard error (SD) of 3 independent measurements using multiple wells each, expressed relative to basal respiration of the control. Quantification of one time‐matched ECAR value in comparison from each metric (Basal, Glycolysis, Capacity and Non‐Glycolytic acidification) is shown below. (C) Intracellular S1P levels of ACM measured by LCMS from controls and mice after 2 weeks 30 μg/L DOP treatment (*n* = 11). (D) Glucose uptake of DOP‐treated ACMs (*n* = 4) compared to controls (*n* = 5) as determined by Glucose Uptake‐Glo Assay. Tracings and quantification of S1P‐stimulated ACMs (*n* = 3) compared to untreated cells (*n* = 3) for Seahorse (E) Glycolytic Rate and (F) Glycolysis Stress assays. Data for C and D are shown as mean ± SD and analysed by Mann–Whitney‐*U*‐test. The Seahorse measurements of A, B, E and F were statistically analysed using a two‐way ANOVA. *p < 0.05, **p < 0.01 and ****p < 0.0001.

To investigate the effect of high intracellular S1P in vivo on ACM glycolysis, we treated mice with the S1P lyase inhibitor 4‐deoxypyridoxine (DOP) for 2 weeks, a treatment that we routinely use to raise S1P concentrations in vivo [45, 46, 47, 48]. These ACMs displayed a significant increase in basal glycolysis and a ~45% higher glycolytic capacity (anaerobic glycolysis) as measured by extracellular acidification rate (ECAR) after rotenone injection compared to non‐treated ACMs in Seahorse Glycolytic Rate assays compared to non‐treated ACMs (Figure 1A). Furthermore, Glycolysis Stress assays demonstrated a 24% increase in ACM glycolysis after exposure to glucose and ~44% higher compensatory anaerobic glycolysis (Figure 1B). We confirmed the increased intracellular S1P levels by DOP as ACM isolated from DOP‐treated mice showed 3‐fold higher S1P content (Figure 1C). To test whether increased glycolysis may be accompanied by increased glucose uptake, we measured 2‐deoxyglucose‐6‐phosphate (2DG6P) incorporation in the Glucose Uptake‐Glo assay and observed that ACM from DOP‐treated mice featured a 3‐fold increase (Figure 1D). These effects were not due to S1P receptor signalling as acute stimulation with S1P had no effect on Glycolytic Rate or Glycolysis Stress (Figure 1E,F).

### 3.2 Stimulation of ACM Glycolysis by S1P Lyase Inhibition Is Dependent on the Insulin‐Sensitive GLUT4 Transporter

**FIGURE 2:**
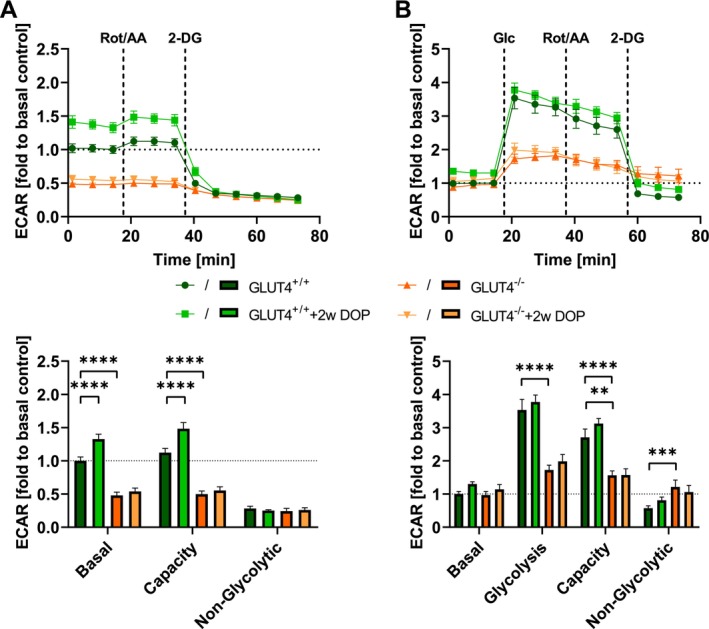
DOP effect is dependent on the insulin‐sensitive GLUT4 transporter. Agilent Seahorse XFe96 Extracellular Flux Analyser was used to analyse and compare (A) the glycolytic rate and (B) glycolysis stress profiles of ACM from GLUT4^−/−^ mice (*n* = 2) and genetic background controls (GLUT4^+/+^, *n* = 5) and investigate the effect of S1P lyase inhibition (*n*_GLUT4+/++2wDOP_ = 3, *n*_GLUT4−/−+2wDOP_ = 3). Trace and quantification of measured extracellular acidification are shown as relative to basal conditions as 1. Statistical differences were calculated using two‐way ANOVA and considered significant with *p* < 0.05. **p < 0.01, ***p < 0.001 and ****p < 0.0001.

To test whether the stimulatory effect on glycolysis was dependent on glucose uptake, we performed the same experiments using DOP in mice with cardiac‐specific deletion of GLUT4, the dominant insulin‐dependent glucose transporter in the heart [49]. Without DOP, Glycolytic Rate revealed a 52% decrease in GLUT4^−/−^ ACM compared to GLUT4^+/+^ ACMs (Figure 2A) and only a 4% increase in GLUT4^−/−^ ECAR in comparison to the 12% in GLUT4^+/+^ (Figure 2B). Glycolysis Stress assays also showed a significant impairment of glucose utilisation in GLUT4^−/−^ versus GLUT4^+/+^ ACM: after glucose administration, the acidification rate of GLUT4^+/+^ ACM rose to 3.53‐fold of baseline, whereas ECAR of GLUT4^−/−^ ACM was only 1.73‐fold higher than baseline (Figure 2B). Most importantly, DOP treatment for 2 weeks had no stimulatory effect on glycolysis whatsoever in GLUT4^−/−^ ACM. Our data suggest that the stimulatory effect of S1P lyase inhibition is due to an increase in glucose uptake by cardiomyocyte GLUT4.

### 3.3 High Fat Diet Impairs Glycolysis in ACM and Causes Subtle Changes in Cardiac Function

**FIGURE 3:**
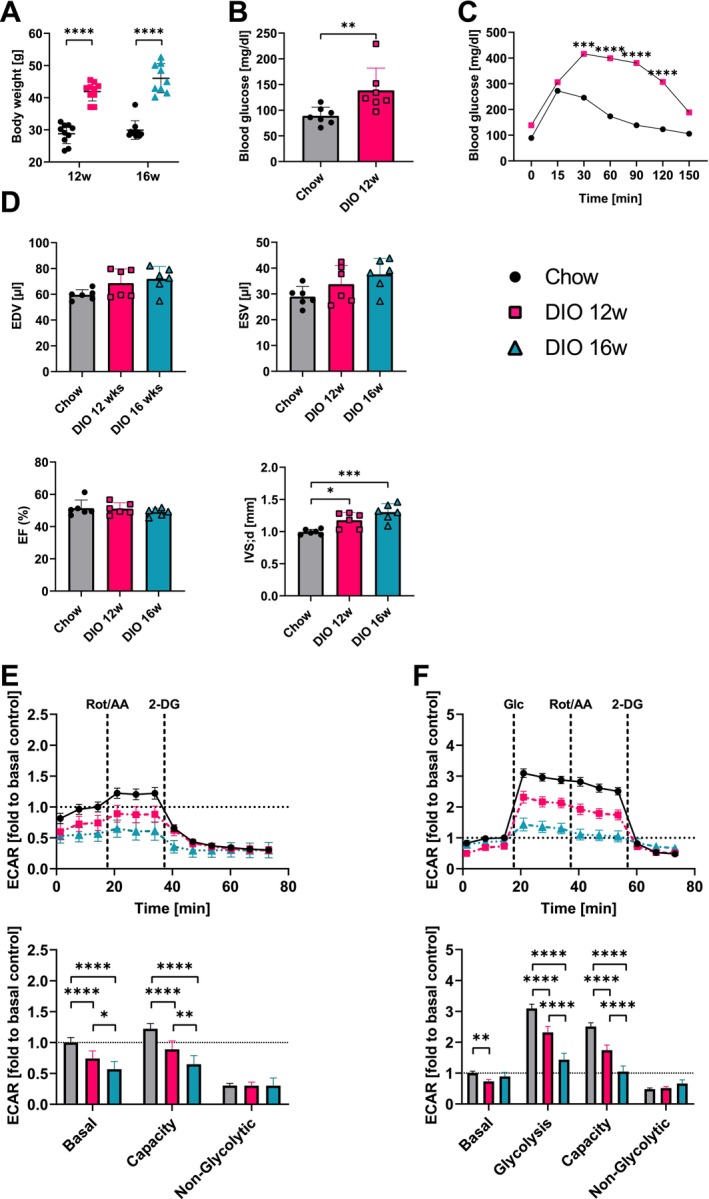
High fat diet leads to time‐dependent deterioration in ACM glucose utilisation. Terminal bodyweight of (A) C57BL/6J mice fed a high‐fat diet (DIO) were compared to normal chow‐fed controls (Chow, *n*_12w_ = 10, *n*_16w_ = 10) after 12 weeks (12w, *n* = 10) and 6 additional weeks (16w, *n* = 9). (B) Fasting blood glucose levels (*n* = 7) and (C) glucose tolerance test in DIO mice compared to chow control mice (*n* = 7). (D) Echocardiography: left ventricular end‐diastolic volume (EDV, μL), end‐systolic volume (ESV, μL), ejection fraction (EF, %), and intraventricular septum thickness (IVS, mm) in DIO (12 and 16w) compared to chow animals (*n*_Chow_ = 6, *n*_12w_ = 6 and *n*_16w_ = 6). Evaluation of the glycolytic phenotype in cardiomyocytes from Chow and DIO (12 and 16w) mice (*n*_Chow_ = 8, *n*_12w_ = 5 and *n*_16w_ = 3) using the Seahorse XF Analyser: (E) Glycolytic Rate assay and (F) Glycolysis Stress Test and Extracellular acidification rate (ECAR) was normalised to baseline and reported for basal glycolysis, glycolytic capacity and non‐glycolytic acidification. *p* value of B was calculated with a Mann–Whitney‐*U*‐test. Statistical analysis of D was performed using one‐way ANOVA, A, C, E and F were analysed using two‐way ANOVA. *p < 0.05, **p < 0.01, ***p < 0.001 and ****p < 0.0001.

We then asked the question of how ACM glucose metabolism is altered in mice rendered insulin‐resistant by diet‐induced obesity (DIO). To answer this, we fed mice a high‐fat diet (HFD) for 12 and 16 weeks and compared glycolysis of isolated ACM to that of age‐matched normal chow‐fed controls—an experiment that, to our knowledge, is the first to examine glycolytic function in prediabetes in isolated primary adult cardiomyocytes using real‐time glycolytic flux measurements. These mice were obese and displayed a pathological glucose tolerance test (Figure 3A–C). Cardiac function was not altered as neither ejection fraction (EF), end‐diastolic volume (EDV), nor end‐systolic volume (ESV) were changed (Figure 3D). However, the interventricular septum (IVS) was thickened after 12 (~18%) and 16 weeks (~30%, Figure 3D) as a sign of beginning hypertrophy.

Very much in contrast to these subtle changes, there were dramatic alterations in glucose metabolism. Glycolytic Rate assays revealed a reduction of basal glycolysis by 25% after 12 weeks and 50% after 16 weeks, respectively, as estimated by the changes in ECAR compared to normal chow‐fed controls (Figure 3E). Furthermore, while ECAR increased by 22% in response to Rot/AA in control ACM as a measure of anaerobic glycolysis, it was significantly impaired at 12 weeks HFD and decreased to only 14% at 16 weeks HFD (Figure 3E). In the Glycolysis Stress assays, ECAR increased by 300% after glucose administration in ACM from normal chow‐fed mice, which was reduced significantly to ~230% at 12 weeks and ~140% at 16 weeks high‐fat DIO (Figure 3F).

### 3.4 S1P Lyase Inhibition After Onset of Glycolytic Defects Completely Restores Glucose Utilisation

**FIGURE 4:**
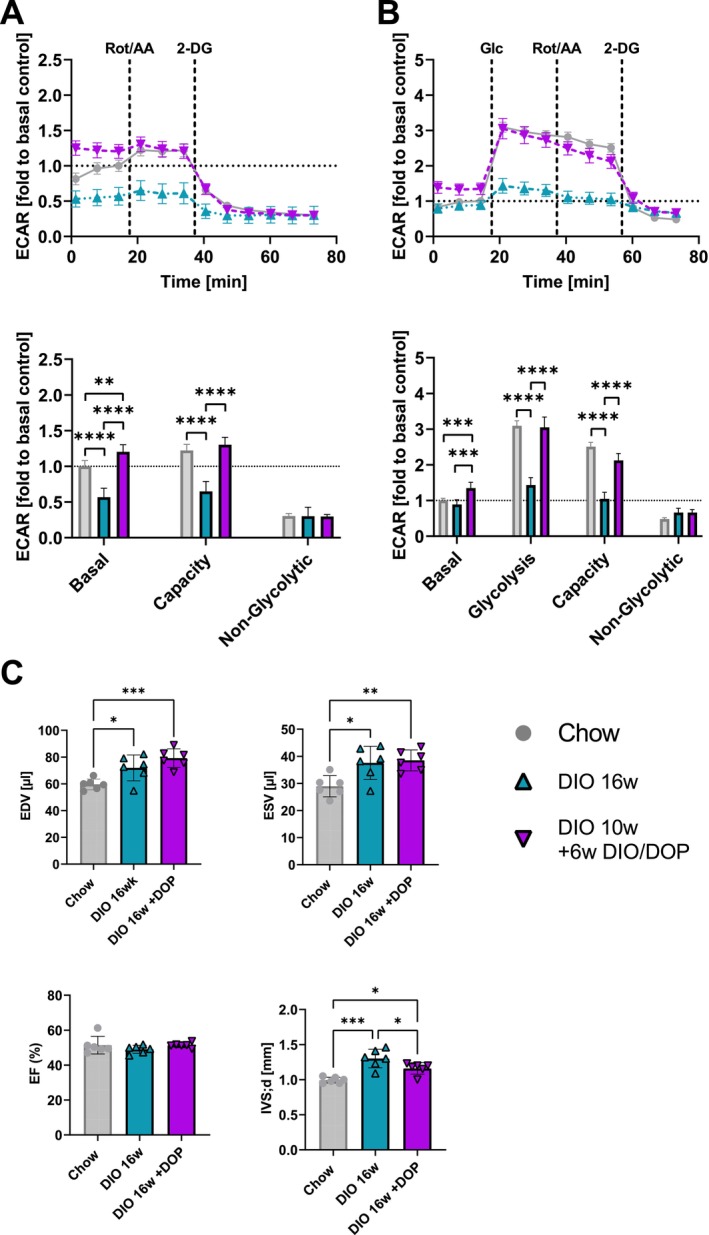
Therapeutic inhibition of S1P lyase restores glucose utilization in diabetic ACM. Tracing of ECAR during Seahorse XF (A) Glycolytic rate and (B) Glycolysis Stress assays from ACM of DIO and DIO+DOP treated mice (*n* = 3) normalised to Chow (grey). Extracellular acidification rate (ECAR) was normalised to baseline and reported for basal glycolysis, glycolytic capacity and non‐glycolytic acidification. (C) Echocardiography: left ventricular end‐diastolic volume (EDV, μL) and end‐systolic volume (ESV, μL), ejection fraction (EF, %), and intraventricular septum thickness (IVS, mm) in Chow, 16 week DIO (*n* = 6), and DIO 10w+6w DIO/DOP (*n* = 6) animals. Significance levels in the Seahorse XF assays A and B were analysed with a two‐way ANOVA and C was analysed using one‐way ANOVA. *p < 0.05, **p < 0.01, ***p < 0.001 and ****p < 0.0001.

To test whether S1P lyase inhibition can improve the dysfunctional glucose utilisation of ACM, we first fed mice a HFD for 10 weeks and then administered DOP for another 6 weeks together with HFD. For comparison, we fed HFD to mice for 16 weeks. This treatment improved blood glucose levels but did not lead to a significant body weight loss (data not shown). We then isolated ACM and analysed glycolysis in Glycolytic Rate and Glycolysis Stress assays. We observed that not only did DOP prevent the deterioration of glycolysis occurring between 10 and 16 weeks, but it completely normalised it to levels of ACM from normal chow‐fed mice (Figure 4A,B). As expected, cardiac functional parameters were unaltered, but the increase in IVS thickness was reduced by DOP (Figure 4C).

## 4 Discussion

S1P has potent cardioprotective properties in myocardial infarction, heart failure and preconditioning [41, 42, 50, 51, 52]. In this study, we adressed whether it affects cardiomyocyte metabolism and, if so, whether this may generally contribute to its beneficial properties in the context of the metabolic changes occurring in diabetic cardiomyopathy. In tumour cells, S1P signalling has been shown to improve glycolysis through binding to its receptors (S1PR1–3) and activation of PI3K/AKT/mTOR [53, 54, 55]. We did not observe increase in glycolytic flux in ACMs through exogenous S1P stimulation, suggesting that S1P receptor activation is not sufficient to improve the glycolysis of ACMs to an extent detectable by extracellular acidification. Our data do not exclude receptor‐mediated effects on glycolysis but show that the S1P/S1PR axis and downstream signal cascades do not convey fast‐acting glycolytic pathways. In our study, inhibition of S1P degradation and elevated levels of endogenous S1P and/or tonic S1P signalling or any other DOP effect also contributed to an improvement in blood glucose levels with unknown cause, although body weight did not change, leaving systemic effects as another possibility of metabolic ACM conditioning. Our experimental setup focused specifically on isolated ACM and revealed enhanced glycolysis. Indeed, improvement of glycolysis as a result of an inactive S1P lyase has been shown in mouse embryonic fibroblasts, where sphingosine‐1‐phosphate lyase 1‐deficient cells presented with increased uptake and metabolisation of glucose [54]. The elevated glucose uptake in these cells was linked to an increased expression level of GLUT1. To investigate if GLUT4, as the major glucose transporter in ACMs, is responsible for the enhanced glycolysis after DOP treatment, we analysed ACMs from mice with selective ablation of GLUT4 in the heart and observed that the DOP effect on glycolysis was absent, leading us to believe that the increased glucose influx involves insulin‐stimulated GLUT4 activation. Indeed, we have observed effects of intracellular S1P on glycolysis in red blood cells through externalisation of GLUT1 and GLUT4 [43]. Furthermore, DIO ACMs had reduced protein levels of GLUT4, phosphorylated AKT1 and insulin receptor that were augmented by lyase inhibition (data not shown), providing a possible regulatory mechanism that remains unknown and is still under investigation.

The development of heart diseases like diabetic cardiomyopathy is commonly linked to the impairment of cardiometabolic homeostasis by insulin resistance, the reduced trafficking of GLUT4 to the cell surface in response to insulin, and impaired glycolysis in favour of a shift towards fatty acid oxidation. Shared aspects between comorbidities and risk factors of cardiovascular diseases like metabolic syndrome and diabetes feature hyperglycaemia, hyperlipidaemia, hyperinsulinaemia. To our knowledge, we are the first to show a rapid deterioration of glycolysis in ACM of diabetic mice that precedes impairment of cardiac function, albeit correlating with mild hypertrophy. Our most important finding was that we could reverse the metabolic depression of glycolysis caused by HFD even after its onset by administration of DOP and ameliorate cardiac hypertrophy, respectively.

Increasing glucose utilisation at early stages of diabetic cardiomyopathy to counteract the cardiomyocyte metabolic derangement caused by hyperglycaemia may offer a new avenue for therapeutic intervention. It aligns with approaches similarly designed to target other metabolic pathways such as reduction of FA levels by inhibiting lipoprotein lipase (Xenical Orlistat) [56, 57, 58], carnitine palmitoyltransferase I inhibitors for reduction of mitochondrial FA import (Pexid Perhexiline) [59, 60, 61] and β‐oxidation (Ranexa Ranolazine) [62, 63], respectively.

## Author Contributions

**Jens Vogt:** conceptualization (equal), data curation (equal), formal analysis (equal), investigation (lead), methodology (equal), validation (equal), visualization (lead), writing – original draft (lead). **Melissa Kim Nowak:** data curation (equal), formal analysis (supporting), investigation (equal), visualization (supporting). **Marcel Benkhoff:** data curation (equal), formal analysis (equal), investigation (equal), visualization (supporting), writing – review and editing (equal). **Hao Hu:** data curation (equal), formal analysis (supporting), investigation (equal). **Philipp Wollnitzke:** investigation (supporting), methodology (equal). **Lisa Dannenberg:** resources (equal). **Amin Polzin:** conceptualization (equal), funding acquisition (equal), project administration (equal), supervision (equal). **Bodo Levkau:** conceptualization (equal), funding acquisition (equal), project administration (equal), supervision (equal), writing – original draft (equal).

## Consent

The authors have nothing to report.

## Conflicts of Interest

The authors declare no conflicts of interest.

## Data Availability

Data will be available from the corresponding author upon reasonable request.
